# A comprehensive and longitudinal evaluation of the different populations of lymphoid and myeloid cells in the peripheral blood of patients treated with chemoradiotherapy for head and neck cancer

**DOI:** 10.1007/s00262-024-03810-6

**Published:** 2024-09-05

**Authors:** Jens von der Grün, Martina Broglie, Matthias Guckenberger, Panagiotis Balermpas

**Affiliations:** 1 Department of Radiation Oncology, University Hospital Zurich, University of Zurich, Universitäts Spital Zürich (USZ), Rämistrasse 100, 8091 Zurich, Switzerland; 2 Department of Otorhinolaryngology-Head and Neck Surgery, University Hospital Zurich, University of Zurich, Zurich, Switzerland

**Keywords:** Chemoradiotherapy, Head and neck cancer, Immunoprofiling, Lymphoid cells, Myeloid cells

## Abstract

**Background:**

Immunotherapy provided significant survival benefits for recurrent and metastatic patients with head and neck cancer. These improvements could not be reproduced in patients treated with curative-intent chemoradiotherapy (CRT) and the optimal radio-immunotherapy (RIT) concepts have yet to be designed. Exploration and analysis of the pre-therapeutic immune status of these patients and the changes occurring during the treatment course could be crucial in rationally designing future combined treatments.

**Methods:**

Blood samples were collected from a cohort of 25 head and neck cancer patients treated with curative-intended (C)-RT prior to therapy, after the first week of treatment, and three months after treatment completion. Peripheral blood mononuclear cells (PBMCs) or all nucleated blood cells were isolated and analyzed via flow cytometry.

**Results:**

At baseline, patients showed reduced monocyte and lymphocyte counts compared to healthy individuals. Although overall CD8^+^ T-cell frequencies were reduced, the proportion of memory subsets were increased in patients. Radiotherapy (RT) treatment led to a further increase in CD8^+^ effector memory T-cells. Among myeloid populations, tumor-promoting subsets became less abundant after RT, in favor of pro-inflammatory cells.

**Conclusion:**

The present study prospectively demonstrated a complex interplay and distinct longitudinal changes in the composition of lymphocytic and myeloid populations during curative (C)-RT of head and neck cancer. Further validation of this method in a larger cohort could allow for better treatment guidance and tailored incorporation of immunotherapies (IT) in the future.

**Supplementary Information:**

The online version contains supplementary material available at 10.1007/s00262-024-03810-6.

## Introduction

Squamous cell carcinoma of the head and neck (SCCHN) accounts for 6% of the total incidence of malignant tumors, and about 500,000 new patients are diagnosed each year around the world [1]. Definitive chemoradiotherapy (CRT) or radical surgery, i.e., resection of the primary tumor and dissection of regional lymph nodes, followed by adjuvant radiotherapy (RT) or CRT, are routinely performed in loco-regionally advanced SCCHN leading to a 5-year overall survival rates of 40–60% [2, 3]. Many advances regarding tumor control and patient survival have been made over the past decades [4].

The advent of immunotherapy (IT) provided significant survival benefits for recurrent and metastatic SCCHN patients for the first time since years [5]. Yet, these improvements could not be reproduced in patients treated with curative-intent CRT [6], and the optimal radio-immunotherapy (RIT) concepts are yet to be designed [7]. Thus, thorough exploration and analysis of the pre-therapeutic immune status of these patients and the changes occurring during the treatment course could be crucial in rationally designing combined treatments in the future.

As an exploratory, ancillary study to a single-center prospective trial evaluating the benefits of adaptive RT for stage II-IVB SCCHN (NCT03972072), venous blood from patients treated within the trial was collected and analyzed using flow cytometry. In the context of CRT for SCCHN, both the innate and adaptive immune system and local tumor infiltrating and peripheral blood immune cells play a key role for patients’ outcome [8, 9]. However, most studies regarding blood-based biomarkers have exclusively assessed only pre-treatment liquid biopsies for their prognostic impact, and only included non-immune biomarkers [9]. A comprehensive profiling of all major immune cell subpopulations was not performed before.

The aim of this pilot study was to longitudinally evaluate different populations of lymphoid and myeloid cells in the peripheral blood of patients treated with definitive (C)-RT for head and neck cancer in order to assess abundances and relative changes of these cell types occurring during the treatment course from baseline to follow-up.

## Methods

### Patient population and treatment

The MARTHA trial is an ongoing, single-center, prospective, open label, single-arm study investigating a possible reduction in late-term toxicity for patients with newly diagnosed and histologically confirmed SCCHN of the oral cavity, oro-, hypopharynx or larynx in the UICC stages II-IVB treated with MR-guided, adaptive standard-dose bilateral (C)-RT. Further, inclusion requires an ECOG Performance score < 2 (Eastern Cooperative Oncology Group) and complete staging including FDG PET-CT-scan (fluorodeoxyglucose positron emission tomography-computed tomography), contrast enhanced magnetic resonance imaging (MRI), or computed tomography (CT) prior to registration. Normo-fractionated RT consists of 2 Gray (Gy) single-dose and 70 Gy cumulative dose to macroscopic tumor (planned target volume (PTV)-70 Gy: primary and involved nodes), 60 Gy to involved nodal levels (PTV-60 Gy) and 50 Gy for elective nodal irradiation (PTV-50 Gy). Concurrently, standard platinum-based chemotherapy (CT) is administered with Cetuximab as an alternative for platinum-ineligible patients.

Treatment is applied with an MRI-Linac (MRIdian®, ViewRay, OH, USA) with a pre-defined image-guided RT and weekly plan adaptation protocol in order to achieve superior normal tissue sparing. The primary endpoint of the study is the rate of radiation-induced xerostomia of grade 2 or worse 12 months after treatment (NCT03972072). The prospective longitudinal evaluation of the peripheral immune cell subpopulations is an ancillary study performed for the first 25 patients included in the trial. Written informed consent for the trial and the present translational project was given by all study participants. The study was approved by the ethics committee of the University Hospital of Zurich, Switzerland (BASEC No. 2019-00993). The results of the peripheral blood analyses at baseline were further compared to those collected from healthy donors.

### Translational analysis

For the translational study, blood from a healthy cohort (HC) was collected within the “Fundamental research project for phenotypical and functional characterization of different leukocyte subsets in healthy and diseased individuals” (PFCL- 1, BASEC No. 2016-01440) which has been reviewed and approved by the competent Swiss authorities and has been carried out in accordance with the current version of the Declaration of Helsinki, the guidelines of Good Clinical Practice, and Swiss legal requirements. The HC was defined as individuals without known active acute or chronic diseases at the time of blood donation.

This study was performed by withdrawing 20 ml of blood in heparin vacutainer tubes (BD Healthcare) from the tumor patient cohort (TC) at pre-defined time points: Prior to therapy (1), after the first week of treatment (2), and three months after treatment completion (3). The blood was processed within one hour of collection, stained and analyzed by flow cytometry in the same day for all three time points.

Peripheral blood mononuclear cells (PBMCs) or nucleated blood cells were isolated using the traditional Ficoll gradient centrifugation or HetaSep according to the manufacturer’s protocol, respectively. Briefly, for Ficoll isolation 10 ml blood was diluted 1:1 with DPBS (Gibco) and layered on 15 ml of Ficoll Paque Plus (GE Healthcare) at a 45° angle. The tubes were centrifugated at 400 g for 20 min Acc:4, Dec:0 at 20 °C. The PBMC layer was collected and washed with DPBS before staining for flow cytometry analysis. For HetaSep isolation, 10 ml blood was mixed with 2 ml HetaSep (Stemcell) and centrifuged at 100 g for 10 min Acc:9, Dec:2 at 20 °C. Upper layer containing nucleated blood cells were collected and washed with DPBS before staining for flow cytometry analysis.

The isolated cells were stained in FACS (fluorescence activated cell sorting) buffer containing the appropriate fluorescently labeled monoclonal antibodies (mAbs) on ice for 20 min (Supplementary Table 6). Cell viability was assessed by staining with Fixable Viability Dye eFluor™ 780 (Invitrogen). Intracellular staining was performed using Foxp3/transcription factor staining buffer set (Invitrogen) following manufacturers’ instructions. Briefly, samples were fix/permed for 1 h at 4 °C and stained with intracellular staining prepared in 1X wash buffer for 30 min at room temperature. Samples were acquired using BD LSR Fortessa on the same day.

### Statistical analysis

Routine blood samples from patients were compared using a paired Wilcoxon test with SPSS (IBM SPSS Statistics, V29.0, Armonk, NY, USA). Flow data was analyzed by manual gating using FlowJo software and R Studio (R Studio: Integrated Development for R. RStudio, PBC, Boston, MA). All samples were gated following the same hierarchy (Supplementary Figs. 1–3), while the individual gates were adjusted for different samples. The population gating was mainly done visually. For certain markers where the distinction between the positive and negative is less clear, such as CD206, CCR5 and CXCR3, fluorescence minus one (FMO) controls were used. Statistics comparing the HC with the patients were done using an unpaired Wilcoxon test, whereas the statistics comparing the patient visits among each other were done using a paired Wilcoxon test. Paired and unpaired tests were normalized and corrected for multiple testing, separately. For all statistical analyses, significance was accepted at the 95% confidence level (*p* < 0.05). For data visualization, Adobe Illustrator (Adobe Inc. Illustrator, V27.3.1, DE, USA) was utilized.

## Results

### Patients’ characteristics, treatment and outcome

Twenty-five patients of the MARTHA trial receiving definitive (C)-RT were included into this study. The majority of the patients were men (80%), and the median age was 61 years. All patients were in good general condition of an ECOG performance status 0 (84%) or 1 (16%). Most patients had a history of smoking (80%) and/or alcohol (72%) abuse. Primary tumors were mostly located in the oropharynx (68%). In total, 44% of the tumors were p16-positive and 16% had bilateral neck node-involvement. All patients underwent primary RT, mostly with concurrent platinum-based CT (Table 1).

**Table 1:** Baseline characteristics

Characteristic	*n* (%)
Total	25 (100)
Sex	
Male	20 (80)
Female	5 (20)
Age	
Median, years (range)	61 (39–75)
Body mass index	
Median (range; kg/m^2^)	24 (19–40)
ECOG performance status	
0	21 (84)
1	4 (16)
History of smoking	
Yes	20 (80)
No	5 (20)
No. pack years of smokers, median (range)	39 (20–100)
History of alcohol abuse	
Yes	18 (72)
No	7 (28)
Tumor site	
Oral cavity	1 (4)
Oropharynx	17 (68)
Hypopharynx	4 (16)
Larynx	3 (12)
p16 Status	
Positive	11 (44)
Negative	14 (56)
Clinical T category	
cT1	1 (4)
cT2	8 (32)
cT3	9 (36)
cT4	7 (28)
Clinical N category	
cN0	7 (28)
cN1 (p16-positive and negative)	7 (28)
cN2 (p16-positive)	1 (4)
cN2a	0 (0)
cN2b	2 (8)
cN2c	3 (12)
cN3a	0 (0)
cN3b	5 (20)
Bilateral neck involvement	
Present	4 (16)
Not present	21 (84)
Clinical M category	
0	25 (100)
1	0 (0)
Primary radiotherapy	
Yes	25 (100)
No	0 (0)
Concurrent chemotherapy	
Yes	23 (92)
No	2 (8)
Chemotherapy regimen	
Cisplatin, high dose	11 (48)
Cisplatin, low dose	9 (39)
Carboplatin/Docetaxel	2 (9)
Cetuximab	1 (4)

*ECOG* Eastern cooperative oncology group, Cisplatin high dose: 100 mg/m^2^ q3w, Cisplatin low dose: 40 mg/m^2^ q1w

After a median follow-up of 25 months (range 11–27), seven (28%) patients were diagnosed with local or distant recurrences. Two (8%) patients had died following tumor progression.

#### Analysis of myeloid cell populations

There was a significant imbalance between the relative amount of myeloid (*p* = 0.004) cell populations when healthy individuals were compared with the SCCHN patients, with tumor patients showing generally increased levels of myeloid cells (Supplementary Fig. 1).

**Fig. 1:**
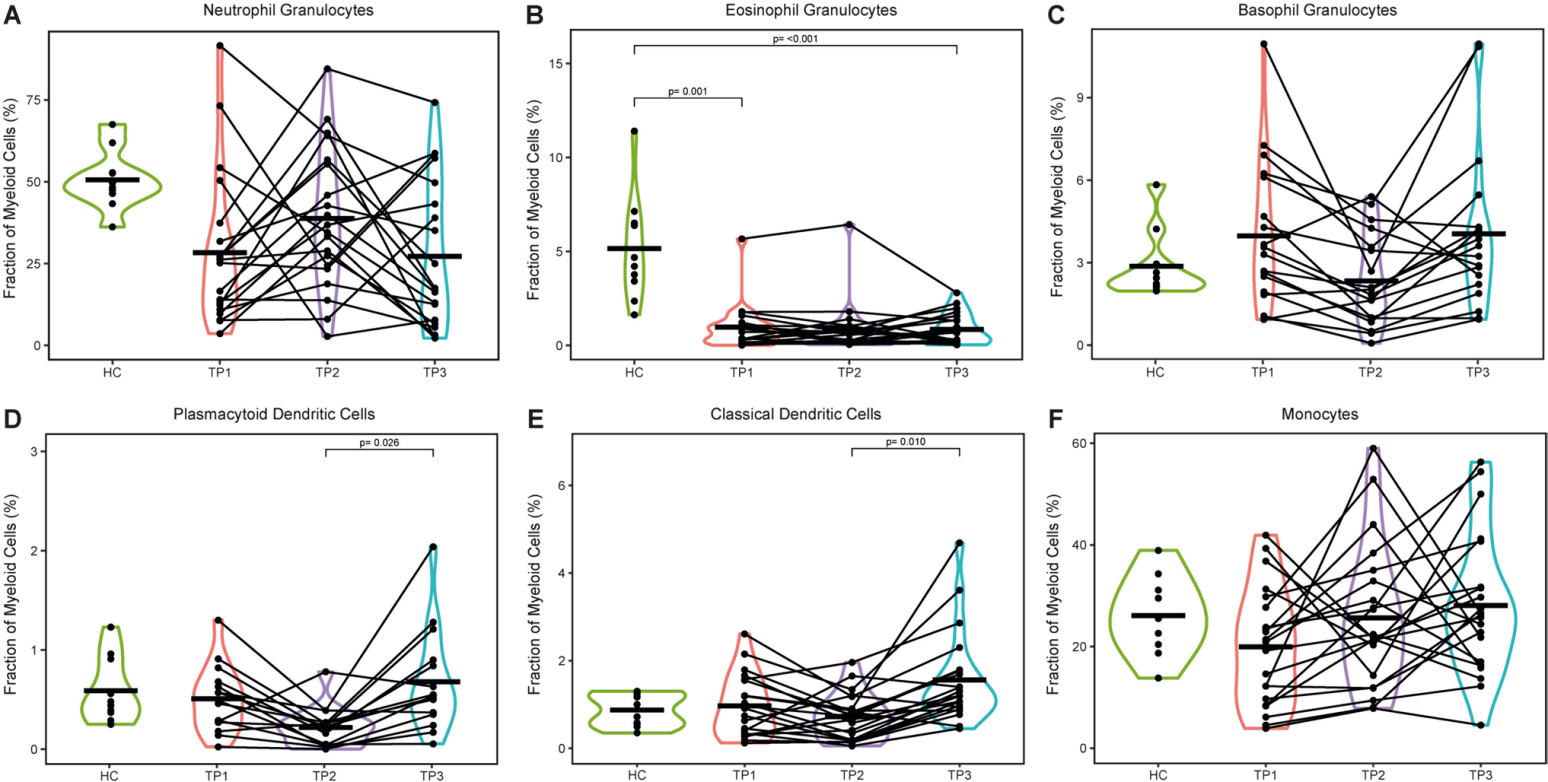
Frequencies of Myeloid Cell Subsets. Abundances of indicated cell subsets were analyzed by flow cytometry and are shown as percentages of myeloid cells. The data of the healthy cohort are compared to three blood collection time points of the tumor patients: TP1: Prior to therapy; TP2: after the first week of treatment; TP3: three months after treatment completion. **a**–**c** Abundances of neutrophil, eosinophil, and basophil granulocytes as percentages of myeloid cells. **d**–**e** Abundances of plasmacytoid and classical dendritic cells as percentages of myeloid cells. **f** Abundances of monocytes as percentages of myeloid cells. *Abbreviations HC* healthy cohort, *TP* time point, *p*: *p* value

Within the myeloid compartment, eosinophil granulocytes were generally less abundant in the TC compared to the HC (*p* < 0.001), while the proportions of neutrophil granulocytes and basophil granulocytes were not significantly altered. The same applied for monocytes. The proportion of classical and plasmacytoid dendritic cells (cDC, pDC) were not changed in comparison with HC and during therapy but their proportion within the myeloid cells were increased during follow-up (*p* = 0.010 and *p* = 0.026, respectively) (Fig. 1).

**Fig. 2:**
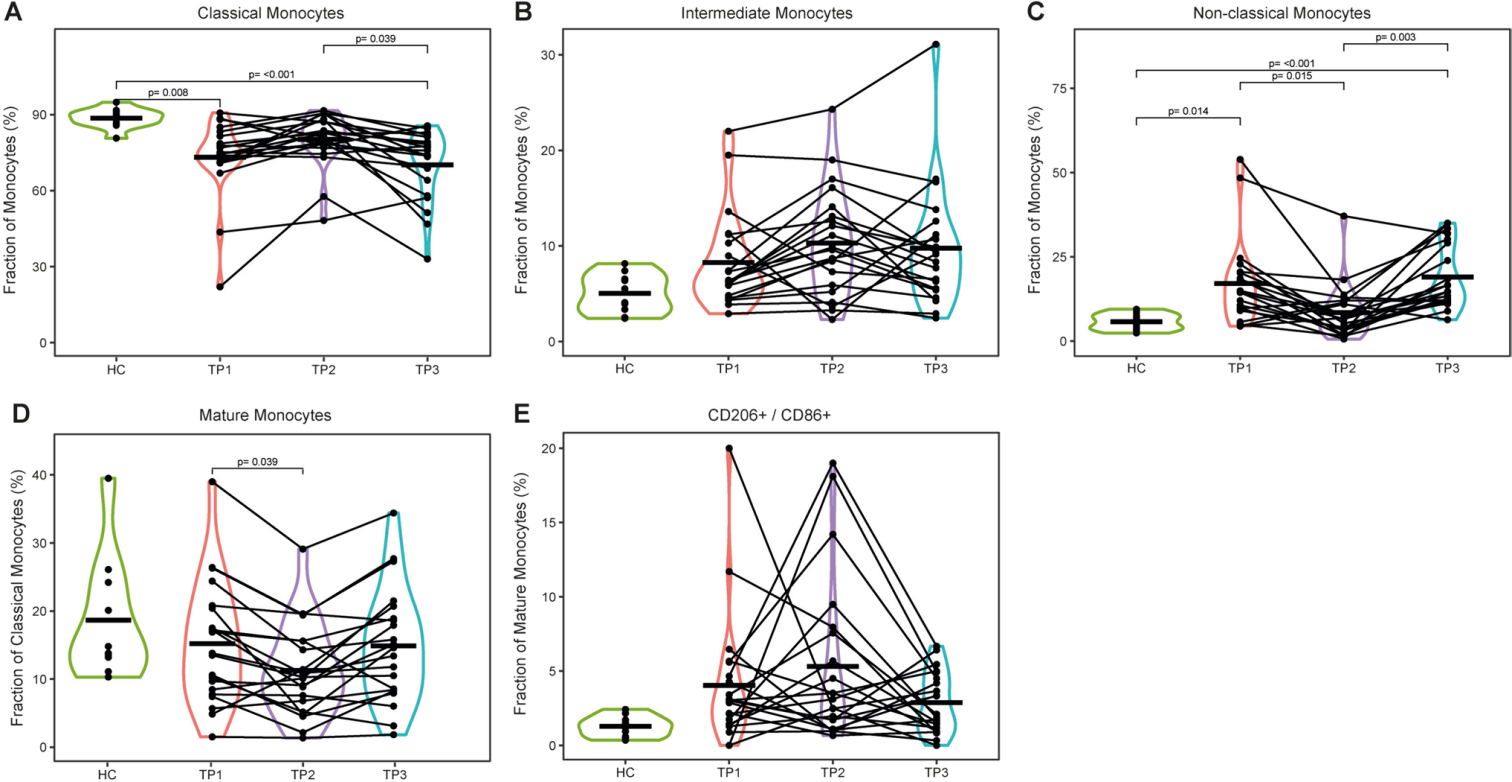
Frequencies of monocyte subsets. Abundances of indicated cell subsets were analyzed by flow cytometry and are shown as percentages of different monocyte fractions. The data of the healthy cohort are compared to three blood collection time points of the tumor patients: TP1: Prior to therapy; TP2: after the first week of treatment; TP3: three months after treatment completion. **a**–**c** frequencies of classical (CD14^+^CD16^−^), intermediate (CD14^+^CD16^+^) and non-classical (CD14^dim^CD16^+^) monocytes as percentages of monocytes. **d** CCR5 expressing, mature monocytes as percentages of classical monocytes. **e** CD86^+^ CD206^+^ cells as percentages of mature monocytes. *Abbreviations HC*: healthy cohort, *TP* time point; *p*: *p* value

Within the monocytes, the proportion of classical monocytes was decreased in the TC compared to the HC (*p* < 0.001). In contrast, non-classical monocytes were more abundant in the TC (*p* < 0.001). Classical monocytes can differentiate into macrophages in tissue and can acquire anti- or pro-tumorigenic phenotypes. CCR5 and CD206 expressions were assessed on monocytes as an indicator of maturation and differentiation into an M2 phenotype, respectively. CD206 expression was more abundant within CCR5^+^ monocytes in the TC compared to the HC, where its expression was virtually absent (Fig. 2, Supplementary Fig. 2).

Regarding the ratio of neutrophil granulocytes to CD3^+^ T lymphocytes, there was no difference when the TC was compared to the healthy individuals or between the three measurements for the TC (Supplementary Fig. 3).

#### Analysis of lymphoid cell populations

**Fig. 3:**
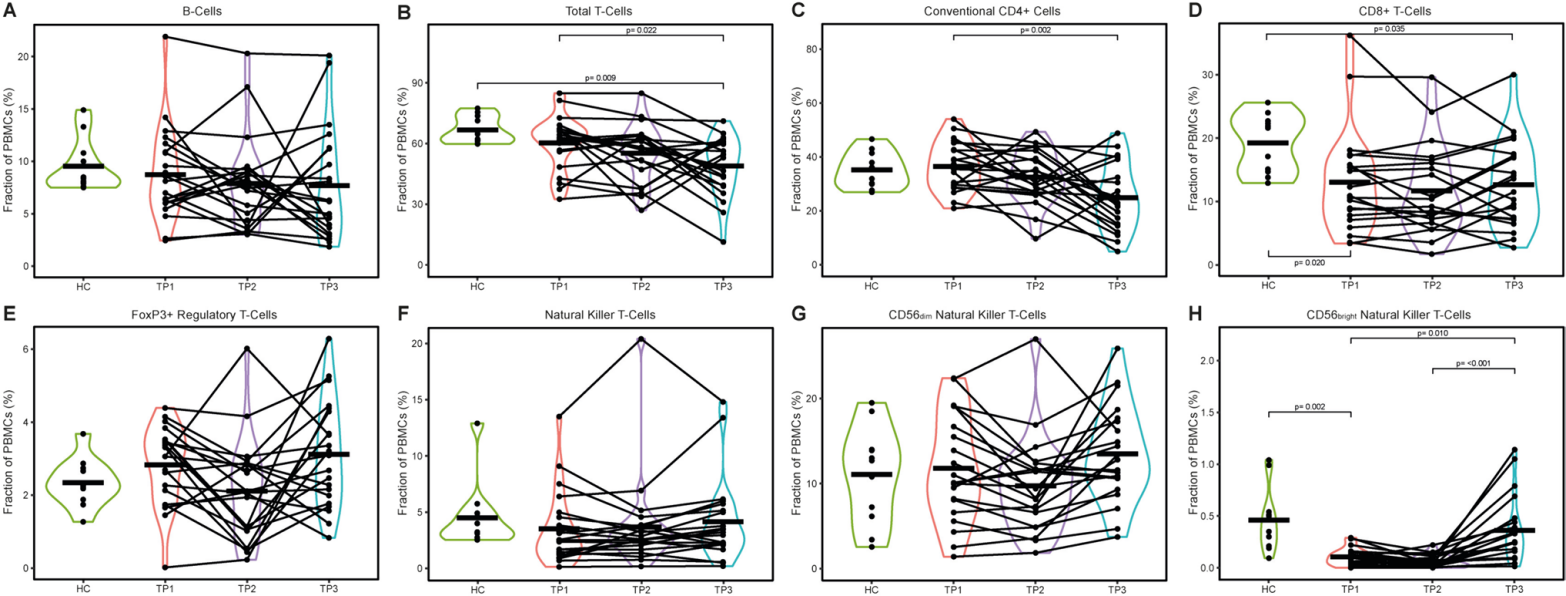
Frequencies of lymphoid cells. Abundances of indicated cell subsets were analyzed by flow cytometry and are shown as percentages of peripheral blood mononuclear cells (PBMCs). The data of the healthy cohort are compared to three blood collection time points of the tumor patients: TP1: Prior to therapy; TP2: after the first week of treatment; TP3: three months after treatment completion. A: Frequencies of B-cells as percentages of PBMCs. B: Frequencies of total T-cells as percentages of PBMCs. C: Frequencies of conventional CD4 + cells as percentages of PBMCs. D: Frequencies of CD8 + T-cells as percentages of PBMCs. E: Frequencies of FoxP3 + regulatory T-cells as percentages of PBMCs. F: Frequencies of natural killer T-cells as percentages of PBMCs. G: Frequencies of CD56-dim natural killer T-cells as percentages of PBMCs. H: Frequencies of CD56-bright natural killer T-cells as percentages of PBMCs. *Abbreviations*: *HC* healthy cohort, *TP* time point, *p*: *p* value; Peripheral blood mononuclear cells: PBMCs

Within the PBMCs, the percentage of total T-cells was declined during therapy and follow-up (*p* = 0.022) which was caused by a reduction in conventional CD4^+^ T-cells (*p* = 0.002). CD8^+^ T-cell percentages were shown to be almost twofold decreased in the TC (*p* = 0.020), which was not altered by therapy. Regarding NK cells, the frequency of CD56^bright^ NK cells was decreased prior to therapy in TC compared to the HC (*p* = 0.002), whereas the frequencies of CD56^dim^ NK cells did not differ. CD56^bright^ NK cells were decreased within the PBMCs also during therapy followed by an increase above the baseline levels following therapy (*p* < 0.001) (Fig. 3, Supplementary Fig. 4).

**Fig. 4:**
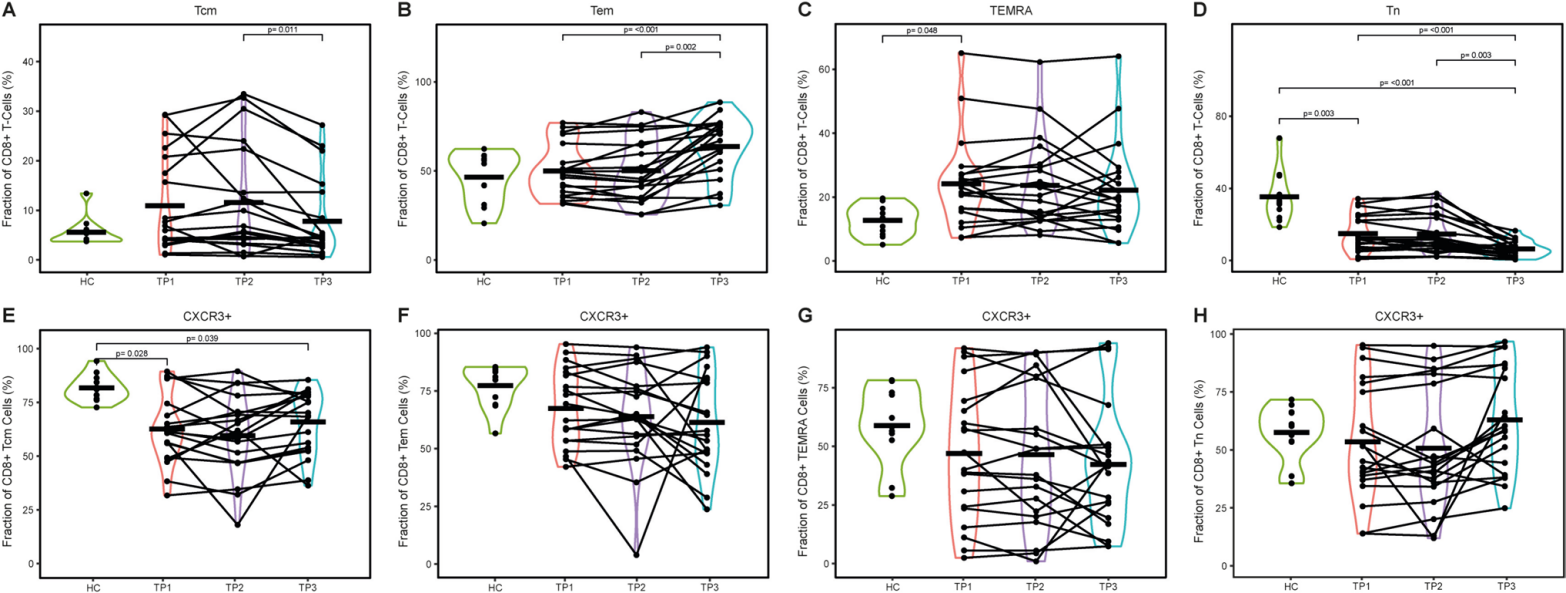
Frequencies of CD8^+^ T-cell subsets. Abundances of indicated cell subsets were analyzed by flow cytometry and are shown as percentages of peripheral blood mononuclear cells (PBMCs). The data of the healthy cohort are compared to three blood collection time points of the tumor patients: TP1: Prior to therapy; TP2: after the first week of treatment; TP3: three months after treatment completion. **a**–**d** Frequencies of central memory (Tcm, CD45RA^−^ CCR7^+^),—effector memory (Tem, CD45RA^−^ CCR7^−^), T-effector memory re-expressing CD45RA (TEMRA, CD45RA^+^ CCR7^−^), and; naïve (Tn, CD45RA^+^ CCR7^+^) CD8^+^ T-cells as percentages of CD8 + T-cells. E–H: Abundance of CXCR3 expressing cells as percentages of CD8 + Tcm, Tem, TEMRA, and Tn cells. *Abbreviations*: Tcm: central memory CD8 + T-cells; Tem: effector memory CD8 + T-cells; TEMRA: T-effector memory re-expressing CD8 + T-cells; Tn: naïve CD8 + T-cells; HC: Healthy cohort; TP: Time point; *p*: *p* value

CD8^+^ T lymphocytes can be further subclassified according to their expression of CCR7 and CD45RA. While naive T-cells (Tn) were generally less abundant in the TC compared to the HC (HC vs. TP1: *p* = 0.003; HC vs. TP1-3: *p* < 0.001), effector cells such as T-effector memory (Tem), T central memory (Tcm) and TEMRA were more abundant in the TC. This remained unchanged during therapy as well as during follow-up (Fig. 4a–d, Supplementary Fig. 5).

Furthermore, for CD8 + T-cells, CXCR3 is a key chemokine receptor required for migration into the tissue. The expression of this marker was reduced for the subset of Tcm cells (HC vs. TP1: *p* = 0.025; HC vs. TP1-3: *p* = 0.039) but unchanged for Tem, Tn, and TEMRA cells in the TC compared to the HC (Fig. 4e–h).

### Quantitative peripheral blood counts from routine tests

Prior to therapy, during RT and at follow-up visits, patients routinely received peripheral blood withdrawals. C-reactive protein (CRP)/albumin ratio was significantly reduced following therapy when compared to the first week of treatment (*p* = 0.023), whereas the ratio of neutrophil/lymphocyte (N/L) significantly increased during and after therapy when compared to the baseline levels (*p* = 0.010 and 0.013). Furthermore, hemoglobin, platelets, and leukocytes were decreased three months after therapy compared to the baseline levels (*p* values: 0.001, < 0.001, and < 0.001, respectively; Supplementary Table 2).

## Discussion

Although there is emerging evidence about the role of both myeloid and lymphoid immune cells in promotion, response to therapy and recurrence of head and neck cancer, most data originate from tumor infiltrating/ -associated populations in the tissue. Little is known both about the possible impact of immune cells in the peripheral blood and their longitudinal changes during CRT [10]. Even less data exist regarding specific subpopulations, other than the commonly studied neutrophil granulocytes and CD4^+^/CD8^+^ lymphocytes. The aim of this exploratory study was not only to provide a detailed descriptive analysis of all the important subpopulations of the innate and adaptive immune system found in the peripheral blood of patients with SCCHN, but also to compare them to those of healthy individuals and identify significant changes during and after standard of care curative-intended treatment. To the best of our knowledge, longitudinal assessments of all major immune cell types from the peripheral blood of patients exclusively treated with CRT are very scarce. Most prior studies assessed either only tissue-associated cells, did not implement different time points or healthy controls, or focused solely on neutrophil granulocytes and lymphocyte count.

The present analysis demonstrated a series of interesting findings:

### Comparison of baseline values to healthy individuals

There was a significant imbalance between the relative amount of lymphocytic and myeloid populations when healthy individuals were compared with the SCCHN patients: Tumor patients showed generally increased levels of myeloid cells and decreased T-lymphoid cells. This can be interpreted as a surrogate for the impaired or altered immunity as a reason or result of tumorigenesis. Interestingly, even the absolute lymphocyte count alone has been shown to be predictive for outcome in patients receiving combined IT and RT before [11]. This has to be taken into account, especially in the case of irradiation of large volumes of peripheral blood, as it is the case with current recommendations for elective RT volumes of the neck, encompassing both carotid arteries [12]. Such “traditional” approaches might hamper the overall treatment effect and have to be critically reconsidered in the future [13]. Importantly, in the present study, all of the patients received a bilateral RT of the neck lymphatics. Nevertheless, the impact of lymphopenia in relationship to the effects of a curative RT treatment without concomitant IT remains controversial. As was shown by some authors before, it is still unknown if RT-induced peripheral/systemic lymphopenia is of any relevance for the patients’ outcome compared to the decreasing cells after RT of regional lymph nodes [14]. It is possible that peripheral lymphopenia is an epiphenomenon and less relevant for treatment outcome.

### Myeloid cells

First, regarding myeloid cells populations, the significant reduction in classical monocytes, in the TC and after the follow-up time point could be an important finding, as these cells or their direct derivatives in the tissue are generally considered immunosuppressive and their numbers inversely correlate with those of CD8^+^ cells [15, 16]. The opposite was the case with non-classical monocytes, whose numbers significantly increased after treatment. Little is known about this cell-type, but some authors suggest a positive role in recruiting CD8^+^ cells and thus promoting tumor cell killing [17]. Intriguingly, no significant findings were observed for the neutrophil granulocytes frequencies, neither when comparing the HC to the SCCHN patients, or after longitudinal assessment, despite the fact, that there was a slight but significant increase in the N/L ratio over time.

*Neutrophile to Lymphocyte ratio*This N/L ratio has been described as a prognostic factor by several authors and even meta-analyses demonstrated an increased hazard ratio for impaired outcome for a ratio of 2–5 [18, 19]. In a previous single-center, but large cohort of patients with laryngeal carcinoma receiving either definitive or adjuvant RT, a higher baseline N/L ratio significantly correlated with an impaired outcome [20]. Our cohort was too small and only few recurrence events were observed for a correlation with oncological endpoints. The median value observed in the SCCHN patients included in this study for all time points exceeded a value of 3. Nevertheless, some significant increase in both the ratio and the relative neutrophil granulocyte numbers observed compared to baseline, during treatment and at the first follow-up could be an artifact, attributed to the higher resistance to CT of neutrophil granulocytes and the delayed recovery of lymphocytes after CT compared to neutrophil granulocytes populations.

### Lymphatic cells

CD8^+^ effector memory cell rates significantly increased during treatment and at follow-up compared to baseline for treated patients, revealing the immunogenic role of RT in promoting an adaptive response [21]. Recently published data elucidate that the exposure of neo-antigens induced by RT lead to priming and recruitment of CD8^+^ T-cells [22, 23]. Based on that, RT can be used synergistically with immunotherapeutic approaches such as adoptive T-cell treatments in solid tumors [24]. Interestingly, the same temporal change was observed for NK cells. CD56^bright^ NK cells, as separately examined here, have different functions than regular NK cells, as they are less cytotoxic and more cytokine producing. However, this specific differentiation has not often been examined in correlation with CRT. NK cell dysfunction is known to promote tumorigenesis and treatment resistance [25–27]. Moreover, NK cells play a key role in RT-induced inflammation and treatment efficacy [28], and there already exist methods to enhance their effects, e.g., via ataxia telangiectasia and Rad3-related inhibitors (ATRi) [29]. On the other hand, the previously observed phenomenon of RT-induced upregulation of T-regulatory cells (Tregs) in the tissue was not significant here in the blood of irradiated patients, indicating that this possible mechanism of acquired resistance to standard treatment is mostly tissue based. Taken together, the kinetics of different lymphocyte populations in peripheral blood and especially those of the NK cells can serve might be an easy-to-assess predictive tool for response after curative-intended (C)-RT. Niu et al. [30] could already demonstrate this in a small cohort of patients with head and neck cancer with a longer follow-up compared to the present cohort. Regarding the observed differences between the HC and TC, already in the first stages of tumorigenesis, the T-cell response can eliminate cancer cells expressing recognized antigens. Nevertheless, some cancer cells may develop alterations to evade the immune system by immunosuppressive mechanisms that hamper the capability of T-cells to recognize and eliminate them [31]. Such immune-resistant cells acquire selection benefits and lead to tumor progression. Moreover, and although no patients with known immunodeficiency syndromes were recruited for this study, a preexisting impairment of the adaptive immune response can also be a reason for cancer development and progression. Patients with deficient T-cell responses or reduced T-cell levels might be in need of non-T-cell-addressing therapies, such as M2 macrophage depletion or NK cell therapies [32]. Both a lower than normal lymphocyte count at baseline and a reduced NK cell activity have been described before for these patients [33, 34]. Upregulation of tumor-promoting/immune suppressive Tregs in the tissue following RT is a well-described phenomenon that could negatively influence the efficacy of treatments [35, 36], also specifically in SCCHN patients [37]. Importantly, a significant and continuous increase in Tregs in the peripheral blood of SCHNN was observed here. Novel approaches investigate the combination of RT and Tregs-depletion as a possible promising treatment method, which can be also combined with immune-checkpoint-inhibition (ICI) to further enhance the effects [38]. In the recurrent/metastatic setting, in patients treated with IT, Gavrielatou et al. demonstrated that assessment of different pre-treatment PBMCs via FACS was able to identify immune cell phenotypes associated with response to ICI [10]. More precisely, CD8^+^ and CD4^+^ cells with stem-like or exhaustion characteristics as well as CD21^−/low^ B-cells were predictive for an improved response. However, this phenomenon might be specific to treatment via ICI [39].

### Limitations

There are limitations of this study. First of all the relatively low number of patients included and the lack of external validation do not allow for more valid statistical analyses. Biomarker establishment for future trials needs robust correlation with oncological outcomes. However, this was impossible in the current study with only seven events and no specific outliers among these seven patients within the significant cell populations. Moreover, there was no possibility either to perform a multivariate analysis with this limited number of events or to define a statistically meaningful cut-off for the parameters, which would then be arbitrary and overfitted. It should be stressed that the purpose of the study was descriptive, as there is a lack of data describing in such detail and longitudinally mode the peripheral immune cells of a homogeneous, curatively treated cohort of HNSCC patients. Secondly, the CT applied could interfere with the results and the longitudinal changes observed. Third, the mere numbers of immune cells counted and their changes do not always represent the same changes in functionality of these cells. Recent studies revealed polymorphisms, including large heterogeneity in the receptors, signaling and function of several subpopulations of the same cell-type, which can only be assessed by more elaborate methods and can further guide treatment individualization [40].

Nevertheless, this is the only study investigating all major immune cell populations in the peripheral blood, in a prospective and longitudinal manner, implementing state-of-the art flow cytometry in a homogeneous cohort of patients treated with curative (C)-RT and compared the findings also with a HC. Previous efforts in this field only included recurrent/metastatic patients without RT or evaluated only some major lymphocytic and/or neutrophil granulocyte populations.

## Conclusion

The present study prospectively demonstrated the complex interplay and distinct longitudinal changes in the composition of all various lymphocytic and myeloid subpopulations during curative (C)-RT of SCCHN. Major findings were the significantly reduced lymphocyte count already at baseline for untreated patients compared to healthy individuals, the impact of RT treatment to enhance the expression of CD8^+^ effector memory cells, and finally the differential response of myeloid populations, with canonical, tumor-promoting myelocytes becoming less abundant following, in favor of non-canonical CD8^+^ recruiting cells. This quick, noninvasive and increasingly simplified approach could allow for a comprehensive evaluation of the patients’ current immune status at every step of a continuous treatment, a better treatment guidance and finally tailored incorporation of immune therapies in the future [41].

## Supplementary Information

Below is the link to the electronic supplementary material.

Supplementary Figure 1: Abundances of total Myeloid Cells. The data of the healthy cohort are compared to three blood collection time points of the tumor patients: TP1: Prior to therapy; TP2: after the first week of treatment; TP3: three months after treatment completion. Abbreviations: HC: Healty cohort; TP: Time point; p: p value. (TIF 4383 kb)

Supplementary Figure 2: Gating Strategies that were used to define Monocytic Cell Populations. (TIF 38428 kb)

Supplementary Figure 3: Ratio of Neutrophil Granulocytes to CD3+ T-cells. Abbreviations: HC: Healty cohort; TP: Time point. (TIF 6399 kb)

Supplementary Figure 4: Gating Strategies that were used to define Lymphocytic Cell Populations. (TIF 37607 kb)

Supplementary Figure 5: Gating Strategies that were used to define Subtypes of CD8+ T-cells. (TIF 37594 kb)

Supplementary file6 (DOCX 16 kb)

## Data Availability

The data that support the findings of this study are available from the corresponding author, PB, upon reasonable request.

## References

[1] Ferlay J et al (2010) Estimates of worldwide burden of cancer in 2008: GLOBOCAN 2008. Int J Cancer 127(12):2893–2917 21351269 10.1002/ijc.25516

[2] Bernier J et al (2004) Postoperative irradiation with or without concomitant chemotherapy for locally advanced head and neck cancer. N Engl J Med 350(19):1945–1952 15128894 10.1056/NEJMoa032641

[3] Cooper JS et al (2004) Postoperative concurrent radiotherapy and chemotherapy for high-risk squamous-cell carcinoma of the head and neck. N Engl J Med 350(19):1937–1944 15128893 10.1056/NEJMoa032646

[4] Chaturvedi AK et al (2013) Worldwide trends in incidence rates for oral cavity and oropharyngeal cancers. J Clin Oncol 31(36):4550–4559 24248688 10.1200/JCO.2013.50.3870 PMC3865341

[5] Burtness B et al (2019) Pembrolizumab alone or with chemotherapy versus cetuximab with chemotherapy for recurrent or metastatic squamous cell carcinoma of the head and neck (KEYNOTE-048): a randomised, open-label, phase 3 study. Lancet 394(10212):1915–1928 31679945 10.1016/S0140-6736(19)32591-7

[6] Lee NY et al (2021) Avelumab plus standard-of-care chemoradiotherapy versus chemoradiotherapy alone in patients with locally advanced squamous cell carcinoma of the head and neck: a randomised, double-blind, placebo-controlled, multicentre, phase 3 trial. Lancet Oncol 22(4):450–462 33794205 10.1016/S1470-2045(20)30737-3

[7] Rückert M et al (2018) Immune modulatory effects of radiotherapy as basis for well-reasoned radioimmunotherapies. Strahlenther Onkol 194(6):509–519 29500551 10.1007/s00066-018-1287-1

[8] Balermpas P et al (2016) CD8+ tumour-infiltrating lymphocytes in relation to HPV status and clinical outcome in patients with head and neck cancer after postoperative chemoradiotherapy: a multicentre study of the German cancer consortium radiation oncology group (DKTK-ROG). Int J Cancer 138(1):171–181 26178914 10.1002/ijc.29683

[9] Budach V, Tinhofer I (2019) Novel prognostic clinical factors and biomarkers for outcome prediction in head and neck cancer: a systematic review. Lancet Oncol 20(6):e313–e326 31162105 10.1016/S1470-2045(19)30177-9

[10] Gavrielatou N et al (2023) B-cell infiltration is associated with survival outcomes following programmed cell death protein 1 inhibition in head and neck squamous cell carcinoma. Ann Oncol 35:340–350 38159908 10.1016/j.annonc.2023.12.011

[11] Chen D et al (2020) Absolute lymphocyte count predicts abscopal responses and outcomes in patients receiving combined immunotherapy and radiation therapy: analysis of 3 phase 1/2 trials. Int J Radiat Oncol Biol Phys 108(1):196–203 32036004 10.1016/j.ijrobp.2020.01.032

[12] Deutsch E et al (2019) Optimising efficacy and reducing toxicity of anticancer radioimmunotherapy. Lancet Oncol 20(8):e452–e463 31364597 10.1016/S1470-2045(19)30171-8

[13] de Kermenguy F et al (2023) Radio-induced lymphopenia in the era of anti-cancer immunotherapy. Int Rev Cell Mol Biol 378:1–30 37438014 10.1016/bs.ircmb.2023.03.002

[14] Telarovic I et al (2022) Radiation-induced lymphopenia does not impact treatment efficacy in a mouse tumor model. Neoplasia 31:100812 35667149 10.1016/j.neo.2022.100812 PMC9168138

[15] Campbell JD et al (2018) Genomic, pathway network, and immunologic features distinguishing squamous carcinomas. Cell Rep 23(1):194-212.e6 29617660 10.1016/j.celrep.2018.03.063 PMC6002769

[16] Sanford DE et al (2013) Inflammatory monocyte mobilization decreases patient survival in pancreatic cancer: a role for targeting the CCL2/CCR2 axis. Clin Cancer Res 19(13):3404–3415 23653148 10.1158/1078-0432.CCR-13-0525 PMC3700620

[17] Padgett LE et al (2023) Nonclassical monocytes potentiate anti-tumoral CD8(+) T cell responses in the lungs. Front Immunol 14:1101497 37426658 10.3389/fimmu.2023.1101497 PMC10325638

[18] Mascarella MA et al (2018) Neutrophil-to-lymphocyte ratio in head and neck cancer prognosis: a systematic review and meta-analysis. Head Neck 40(5):1091–1100 29356179 10.1002/hed.25075

[19] Takenaka Y et al (2018) Prognostic role of neutrophil-to-lymphocyte ratio in head and neck cancer: a meta-analysis. Head Neck 40(3):647–655 29076207 10.1002/hed.24986

[20] Cichowska-Cwalińska, N., et al., *Radiotherapy-induced dynamic changes in the lymphocyte-to-monocyte ratio in patients with laryngeal cancer indicate poor prognosis.* Frontiers in Oncology, 2023. **13**. 10.3389/fonc.2023.1234953 PMC10598385 37886164

[21] Sharabi AB et al (2015) Radiation and checkpoint blockade immunotherapy: radiosensitisation and potential mechanisms of synergy. Lancet Oncol 16(13):e498-509 26433823 10.1016/S1470-2045(15)00007-8

[22] Formenti SC et al (2018) Radiotherapy induces responses of lung cancer to CTLA-4 blockade. Nat Med 24(12):1845–1851 30397353 10.1038/s41591-018-0232-2 PMC6286242

[23] Formenti SC, Demaria S (2009) Systemic effects of local radiotherapy. Lancet Oncol 10(7):718–726 19573801 10.1016/S1470-2045(09)70082-8 PMC2782943

[24] Laurent P-A et al (2023) Radiotherapy as a means to increase the efficacy of T-cell therapy in solid tumors. OncoImmunology 12(1):2158013 36567802 10.1080/2162402X.2022.2158013 PMC9788698

[25] Trefny MP et al (2020) PD-1+ natural killer cells in human non-small cell lung cancer can be activated by PD-1/PD-L1 blockade. Cancer Immunol Immunother 69(8):1505–1517 32296919 10.1007/s00262-020-02558-z PMC11027624

[26] Trefny MP et al (2019) A variant of a killer cell immunoglobulin-like receptor is associated with resistance to PD-1 blockade in lung cancer. Clin Cancer Res 25(10):3026–3034 30765392 10.1158/1078-0432.CCR-18-3041

[27] Stangl S et al (2018) Heat shock protein 70 and tumor-infiltrating NK cells as prognostic indicators for patients with squamous cell carcinoma of the head and neck after radiochemotherapy: a multicentre retrospective study of the German Cancer Consortium Radiation Oncology Group (DKTK-ROG). Int J Cancer 142(9):1911–1925 29235112 10.1002/ijc.31213 PMC5873418

[28] Walle T et al (2022) Radiotherapy orchestrates natural killer cell dependent antitumor immune responses through CXCL8. Sci Adv. 10.1126/sciadv.abh4050 35319989 10.1126/sciadv.abh4050 PMC8942354

[29] Patin EC et al (2022) Harnessing radiotherapy-induced NK-cell activity by combining DNA damage–response inhibition and immune checkpoint blockade. J Immunother Cancer 10(3):e004306 35314434 10.1136/jitc-2021-004306 PMC8938703

[30] Niu M et al (2021) Comparison of the composition of lymphocyte subpopulations in non-relapse and relapse patients with squamous cell carcinoma of the head and neck before, during radiochemotherapy and in the follow-up period: a multicenter prospective study of the German Cancer Consortium Radiation Oncology Group (DKTK-ROG). Radiat Oncol 16(1):141 34332614 10.1186/s13014-021-01868-5 PMC8325802

[31] Kallingal A et al (2023) Cancer immune escape: the role of antigen presentation machinery. J Cancer Res Clin Oncol 149(10):8131–8141 37031434 10.1007/s00432-023-04737-8 PMC10374767

[32] Greene S, Patel P, Allen CT (2019) How patients with an intact immune system develop head and neck cancer. Oral Oncol 92:26–32 31010619 10.1016/j.oraloncology.2019.03.010 PMC6481300

[33] Kuss I et al (2004) Decreased absolute counts of T lymphocyte subsets and their relation to disease in squamous cell carcinoma of the head and neck. Clin Cancer Res 10(11):3755–3762 15173082 10.1158/1078-0432.CCR-04-0054

[34] Dasgupta S et al (2005) Inhibition of NK cell activity through TGF-beta 1 by down-regulation of NKG2D in a murine model of head and neck cancer. J Immunol 175(8):5541–5550 16210663 10.4049/jimmunol.175.8.5541

[35] Kachikwu EL et al (2011) Radiation enhances regulatory T cell representation. Int J Radiat Oncol Biol Phys 81(4):1128–1135 21093169 10.1016/j.ijrobp.2010.09.034 PMC3117954

[36] Price JG et al (2015) CDKN1A regulates Langerhans cell survival and promotes Treg cell generation upon exposure to ionizing irradiation. Nat Immunol 16(10):1060–1068 26343536 10.1038/ni.3270 PMC4620552

[37] Schuler PJ et al (2013) Effects of adjuvant chemoradiotherapy on the frequency and function of regulatory T cells in patients with head and neck cancer. Clin Cancer Res 19(23):6585–6596 24097865 10.1158/1078-0432.CCR-13-0900 PMC3855337

[38] Ji D et al (2020) Combination of radiotherapy and suppression of Tregs enhances abscopal antitumor effect and inhibits metastasis in rectal cancer. J Immunother Cancer. 10.1136/jitc-2020-000826 33106387 10.1136/jitc-2020-000826 PMC7592256

[39] Miller BC et al (2019) Subsets of exhausted CD8+ T cells differentially mediate tumor control and respond to checkpoint blockade. Nat Immunol 20(3):326–336 30778252 10.1038/s41590-019-0312-6 PMC6673650

[40] Cillo AR et al (2020) Immune landscape of viral- and carcinogen-driven head and neck cancer. Immunity 52(1):183-199.e9 31924475 10.1016/j.immuni.2019.11.014 PMC7201194

[41] Donaubauer A-J et al (2020) Chapter twenty—analysis of the immune status from peripheral whole blood with a single-tube multicolor flow cytometry assay. In: Galluzzi L, Rudqvist N-P (eds) Methods in enzymology. Academic Press, Cambridge, pp 389–415 10.1016/bs.mie.2019.03.003 32000906

