# Supplementary figures and images for: A comprehensive and longitudinal evaluation of the different populations of lymphoid and myeloid cells in the peripheral blood of patients treated with chemoradiotherapy for head and neck cancer
Source: Cancer Immunol Immunother. 2024 Sep 5;73(11):222. doi: 10.1007/s00262-024-03810-6 (PMC11377404; doi:10.1007/s00262-024-03810-6)

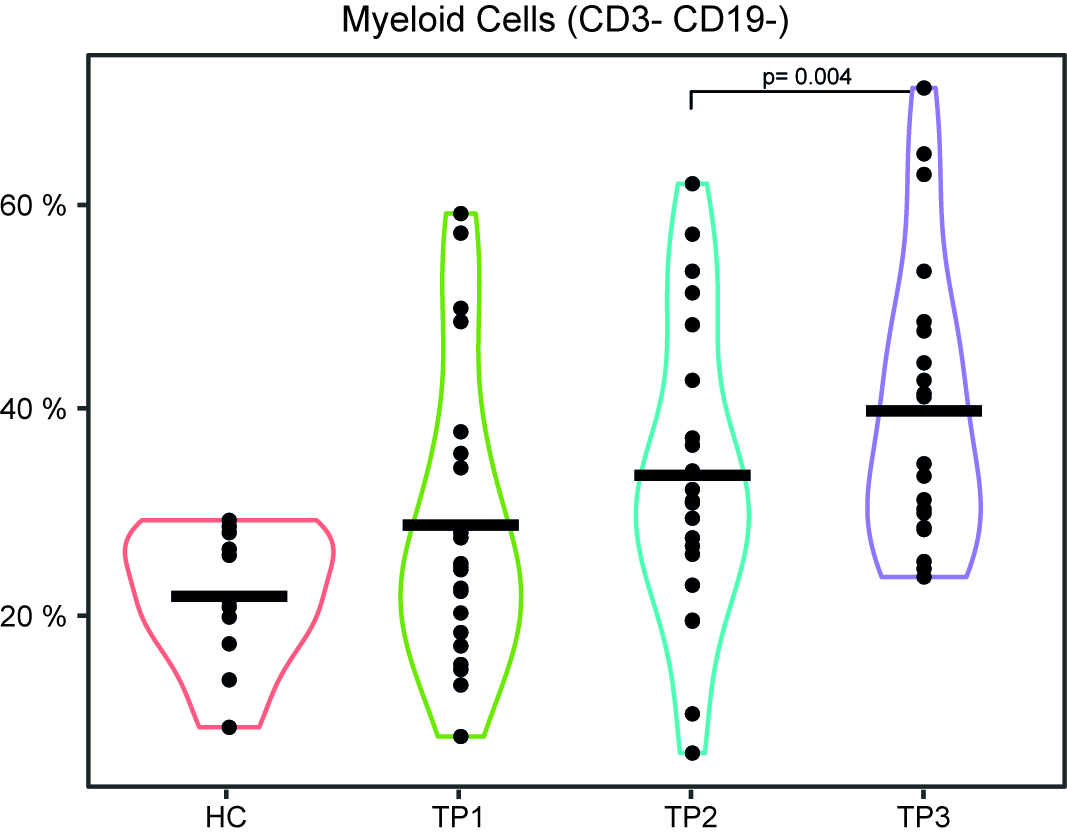

Supplement: Supplementary file 1 — Supplementary Figure 1: Abundances of total Myeloid Cells. The data of the healthy cohort are compared to three blood collection time points of the tumor patients: TP1: Prior to therapy; TP2: after the first week of treatment; TP3: three months after treatment completion. Abbreviations: HC: Healty cohort; TP: Time point; p: p value. (TIF 4383 kb) [file 262_2024_3810_MOESM1_ESM.tif]

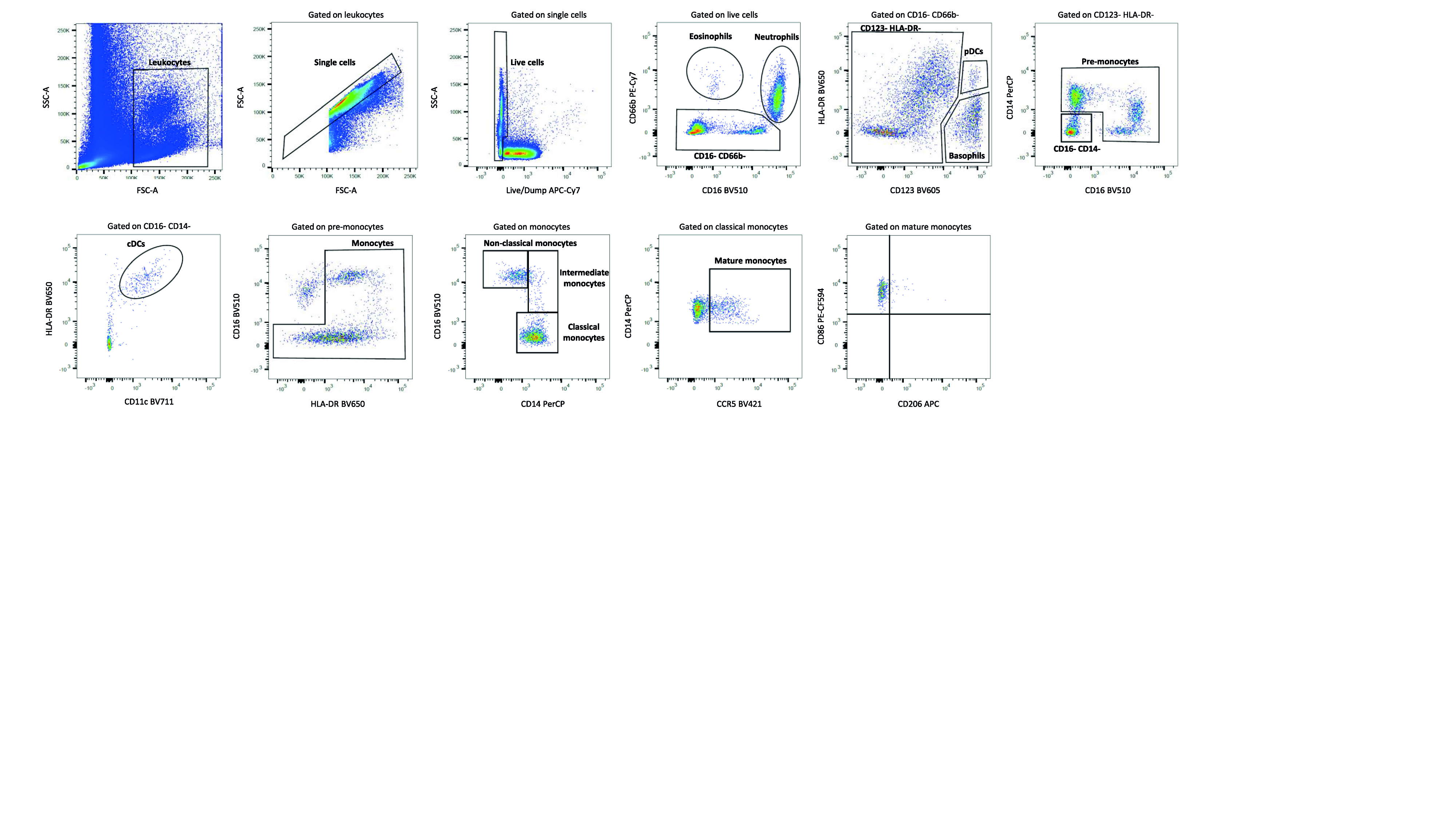

Supplement: Supplementary file 2 — Supplementary Figure 2: Gating Strategies that were used to define Monocytic Cell Populations. (TIF 38428 kb) [file 262_2024_3810_MOESM2_ESM.tif]

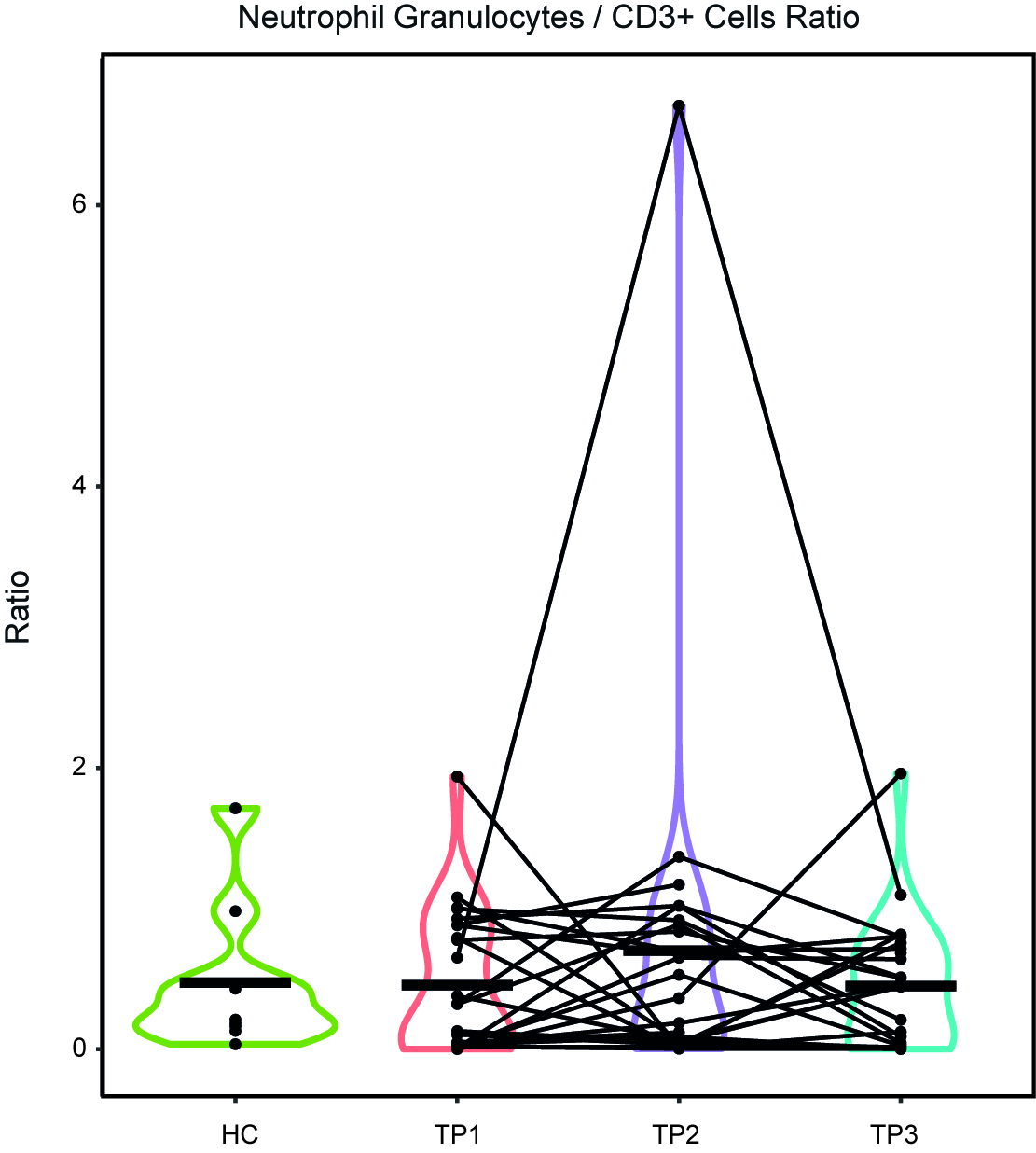

Supplement: Supplementary file 3 — Supplementary Figure 3: Ratio of Neutrophil Granulocytes to CD3+ T-cells. Abbreviations: HC: Healty cohort; TP: Time point. (TIF 6399 kb) [file 262_2024_3810_MOESM3_ESM.tif]

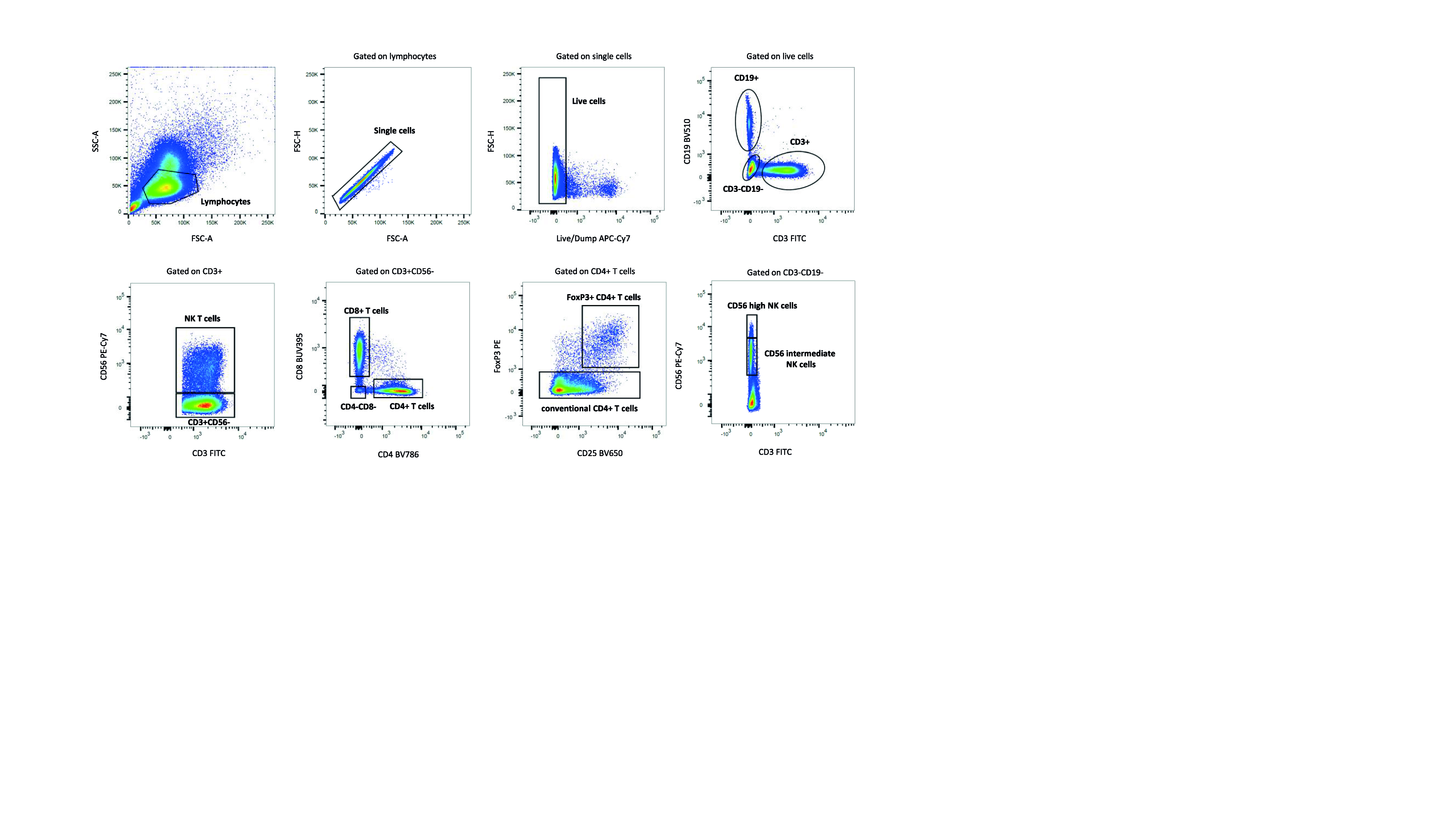

Supplement: Supplementary file 4 — Supplementary Figure 4: Gating Strategies that were used to define Lymphocytic Cell Populations. (TIF 37607 kb) [file 262_2024_3810_MOESM4_ESM.tif]

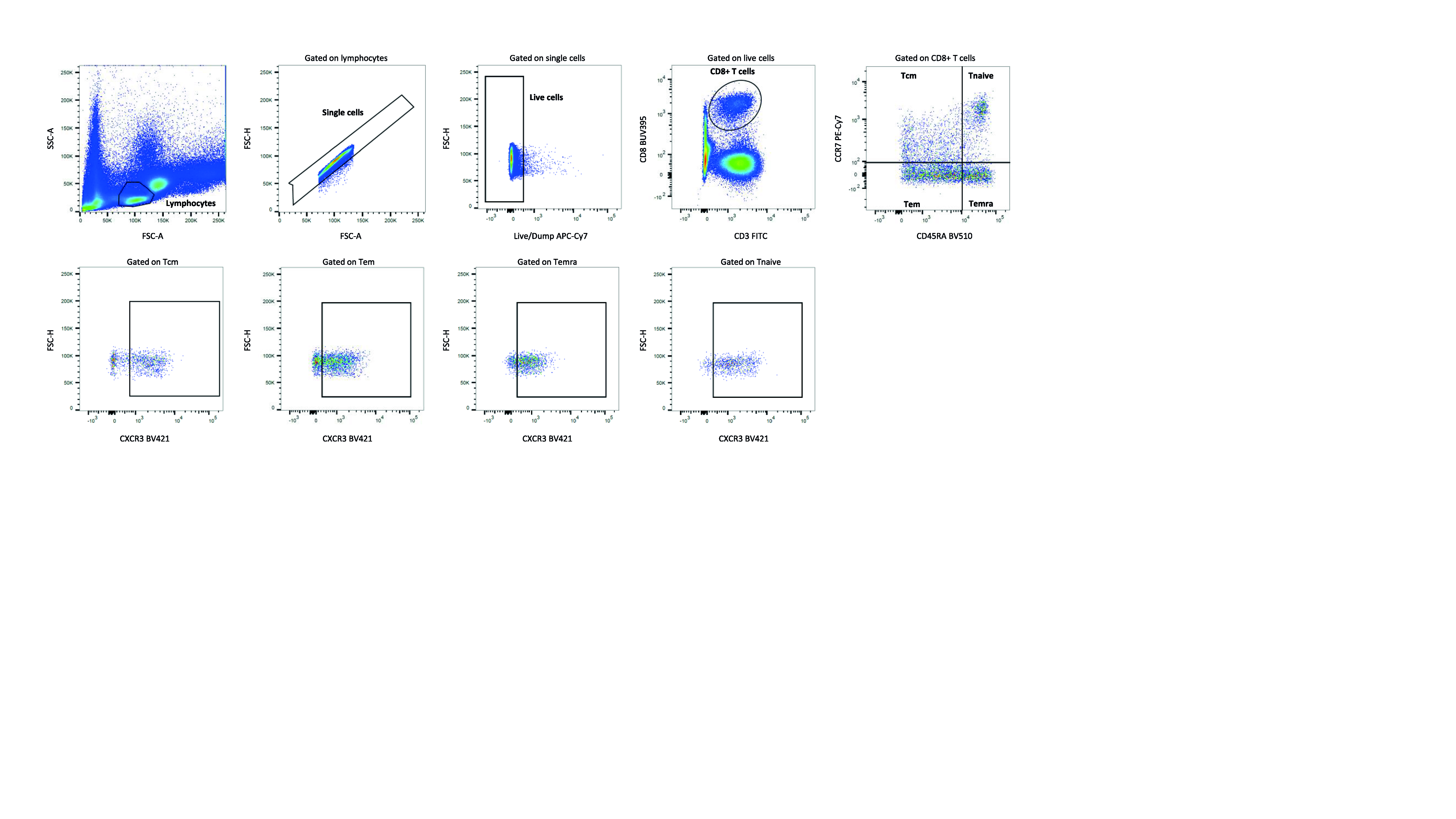

Supplement: Supplementary file 5 — Supplementary Figure 5: Gating Strategies that were used to define Subtypes of CD8+ T-cells. (TIF 37594 kb) [file 262_2024_3810_MOESM5_ESM.tif]
