# Supplementary material for: A comprehensive and longitudinal evaluation of the different populations of lymphoid and myeloid cells in the peripheral blood of patients treated with chemoradiotherapy for head and neck cancer
Source: Cancer Immunol Immunother. 2024 Sep 5;73(11):222. doi: 10.1007/s00262-024-03810-6 (PMC11377404; doi:10.1007/s00262-024-03810-6)
Supplement: Supplementary file 6 — Supplementary file6 (DOCX 16 kb) [file 262_2024_3810_MOESM6_ESM.docx]

**Supplementary Tables**

**Supplementary table 1: Antibodies used for flow cytometry.**

| **Marker** | **Color** | **Company** | **Catalog number** |
| --- | --- | --- | --- |
| CD11c | BV711 | Biolegend | 301630 |
| CD14 | PerCP | Biolegend | 301847 |
| CD16 | BV510 | Biolegend | 302047 |
| CD19 | APC-Cy7 | Biolegend | 302217 |
| CD19 | BV510 | Biolegend | 302241 |
| CD122 | BV421 | Biolegend | 339009 |
| CD123 | BV605 | Biolegend | 306025 |
| CD132 | APC | Biolegend | 338607 |
| CD152 (CTLA-4) | BV785 | Biolegend | 369623 |
| CD183 (CXCR3) | BV421 | Biolegend | 353716 |
| CD195 (CCR5) | BV421 | Biolegend | 359117 |
| CD197 (CCR7) | PE-Cy7 | Biolegend | 353225 |
| CD25 | BV650 | Biolegend | 302633 |
| CD206 | APC | Biolegend | 321109 |
| CD223 (LAG-3) | BV711 | Biolegend | 369319 |
| CD235a | APC-Cy7 | Biolegend | 349115 |
| CD274 (PD-L1) | FITC | Biolegend | 374509 |
| CD279 (PD-1) | APC | Biolegend | 329907 |
| CD3 | FITC | Biolegend | 300305 |
| CD3 | APC-Cy7 | Biolegend | 300317 |
| CD33 | BV785 | Biolegend | 303427 |
| CD366 (TIM-3) | PE | Biolegend | 345005 |
| CD4 | BV785 | Biolegend | 317441 |
| CD45RA | BV510 | Biolegend | 304141 |
| CD56 | APC-Cy7 | Biolegend | 362511 |
| CD56 | PE-Cy7 | Biolegend | 362510 |
| CD66b | PE-Cy7 | Biolegend | 305115 |
| CD8 | PerCP | Biolegend | 344708 |
| CD86 | PE-Dazzle | Biolegend | 305433 |
| FoxP3 | PE | Biolegend | 320107 |
| HLA-DR | BV650 | Biolegend | 307649 |
| Ki-67 | BV711 | Biolegend | 350515 |
| LOX1 | PE | Biolegend | 358603 |

**Supplementary table 2: Peripheral Blood Cell Counts and Values.** Time point 1: Baseline blood values prior to therapy; Time point 2: One week after start of radiotherapy; Time point 3: Three months after completion of radiotherapy; Abbreviation: TP: Time point.

| **Characteristic** | **TP1,** Median (Range) | **TP2,** Median (Range) | **TP3,** Median (Range) | **P-value**  TP1 vs TP2 | **P-value**  TP2 vs TP3 | **P-value**  TP1 vs TP3 |
| --- | --- | --- | --- | --- | --- | --- |
| **Blood Values** |  |  |  |  |  |  |
| **CRP (mg/l)** | 4.00 (0.30-79.00) | 4.40 (0.90-67.00) | 1.70 (0.60-17.00) | 0.605 | **0.032** | 0.073 |
| **Albumin (g/l)** | 43 (36-48) | 42 (31-48) | 44 (39-51) | **0.004** | **<0.001** | 0.871 |
| **CRP/Albumin ratio** | 0.09 (0.01-1.98) | 0.10 (0.01-1.96) | 0.03 (0.01-0.37) | 0.615 | **0.023** | 0.051 |
| **Blood cell counts (G/l)** |  |  |  |  |  |  |
| **Hemoglobin** | 142 (105-170) | 142 (89-161) | 132 (108-163) | **0.017** | **0.028** | **0.001** |
| **Platelets** | 288 (175-484) | 292 (167-528) | 225 (141-343) | 0.798 | **0.001** | **<0.001** |
| **Leukocytes** | 8.52 (5.72-18.26) | 8.77 (4.67-14.30) | 5.26 (2.03-8.04) | 0.893 | **<0.001** | **<0.001** |
| **Differential blood cell counts (G/l)** |  |  |  |  |  |  |
| **Neutrophils** | 5.95 (3.29-13.46) | 6.27 (3.10-13.25) | 3.57 (1.36-5.77) | 0.511 | **<0.001** | **<0.001** |
| **Monocytes** | 0.71 (0.39-1.46) | 0.69 (0.32-1.44) | 0.55 (0.30-0.89) | 0.948 | **0.001** | **<0.001** |
| **Eosinophils** | 0.12 (0.00-0.64) | 0.09 (0-00-0.42) | 0.10 (0.03-0.51) | **0.032** | 0.753 | 0.091 |
| **Basophils** | 0.06 (0.00-0.12) | 0.03 (0.01-0.10) | 0.03 (0.00-0.10) | **0.004** | 0.415 | **0.001** |
| **Lymphocytes** | 1.71 (0.64-3.04) | 1.29 (0.40-2.68) | 0.65 (0.28-1.53) | **0.003** | **<0.001** | **<0.001** |
| **Immature granulocytes** | 0.04 (0.01-0.08) | 0.04 (0.01-0.13) | 0.02 (0.00-0.05) | **0.008** | **<0.001** | **0.003** |
| **Nucleated red blood cells (NRBC)** | 0.00 (0.00-0.00) | 0.00 (0.00-0.01) | 0.00 (0.00-0.00) | **0.025** | **0.025** | 1.000 |
| **Neutrophils/Lymphocytes ratio** | 3.20 (1.35-11.47) | 4.53 (1.67-33.13) | 4.62 (1.46-11.88) | **0.010** | 0.925 | **0.013** |
